# Natural and anthropogenic impact on the microclimate and particulate matter in the UNESCO show cave

**DOI:** 10.1007/s11356-024-34366-8

**Published:** 2024-07-19

**Authors:** Miloš Miler, Nina Zupančič, Stanka Šebela, Simona Jarc

**Affiliations:** 1 Geological Survey of Slovenia, Dimičeva Ulica 14, 1000 Ljubljana, Slovenia; 2 Faculty of Natural Sciences and Engineering, Department of Geology, University of Ljubljana, Aškerčeva 12, 1000 Ljubljana, Slovenia; 3 ZRC SAZU, Ivan Rakovec Institute of Palaeontology, Novi Trg 2, 1000 Ljubljana, Slovenia; 4 ZRC SAZU, Karst Research Institute, Titov Trg 2, 6230 Postojna, Slovenia

**Keywords:** Particle identification, SEM/EDS, Source apportionment, Provenance, Time series analyses, Škocjan Caves

## Abstract

Particulate matter concentrations (PM_10_, PM_2.5_, PM_1_) and microclimatic parameters (air temperature, CO_2_) were monitored in the Škocjan Caves (Slovenia). The effects of tourist visits on the PM concentrations and the cave’s microclimate are immediate and direct, but these values normalise relatively quickly. The results showed seasonal, diurnal, and spatial differences in all parameters studied. Due to the higher number of visitors, their influence on the cave’s microclimate and PM_10_ and PM_2.5_ concentrations is greater in summer than in winter. The measured PM_1_ levels depend on the ventilation in the cave, as air transport plays an important role in their introduction into the cave. PM consists of minerals of natural origin resulting from the re-suspension of cave sediments due to strong air currents generated by the opening of the doors to tourists and their walks. The second most common influence is the anthropogenic phases originating from maintenance work in the cave, electronic devices, cave lighting and emissions from outside the cave (aerosols from the polluted Reka River, industry, traffic, gypsum waste disposal). In order to upgrade the sustainable use of the UNESCO-listed Škocjan Caves for tourism, we propose regular monitoring of PM and a detailed characterization of individual PMs and their sources, in addition to monitoring of the cave’s microclimate and biology.

**Supplementary Information:**

The online version contains supplementary material available at 10.1007/s11356-024-34366-8.

## Introduction

With their specific landforms, especially caves, karst areas usually represent an interface of various environmental compartments and important environmental indicators. Therefore, they are vulnerable ecosystems as they lack self-cleaning capacity, have particular hydrological characteristics, and are prone to rapid displacement of pollutants through karst systems (Kovačič 2003; Zwahlen 2004, van Beynen 2011). Due to their distinctness, caves represent outstanding natural and tourist destinations. Since the first visits to caves, humans have exerted a significant influence on cave environments and ecosystems, and karst terrains are particularly sensitive to their direct or indirect impacts (Brinkmann and Garren 2011; Fernandez-Cortes et al. 2011; Goldscheider 2019). In the caves, which are regularly visited by large groups of tourists, the most common and pronounced changes are those in air temperature, humidity and CO_2_ concentrations, followed by changes in the physicochemical properties of drip-water and the growth of lampenflora, which have a significant impact on the growth of speleothems (e.g. Pulido-Bosch et al. 1997; Gázquez et al. 2016; Moldovan et al. 2020; Constantin et al. 2021). Less studied is the problem of the introduction of aerosols (Mulec et al. 2023; Martin-Pozas et al 2024), including particulate matter (PM). PMs can physically affect the cave environment, particularly their aesthetic value (i.e. expressed as changes in colour) of speleothems (e.g. Liu et al. 2011; Dredge et al. 2013 and references therein). A strong relationship between cave ventilation (both natural and artificial to accommodate the demands of tourism), cave aerosols of inorganic and organic origin and their consequent spatial distribution was confirmed in some caves (Kertész et al. 1999; Dredge et al. 2013; Faimon et al. 2019). Concentrations of aerosols were found to be a function of distance from the cave entrance and active water channels in some caves (Kertész et al. 1999; Smith et al. 2013). It was also demonstrated that under normal continuous speleothem growth and related environmental conditions, aerosol deposition is of minor importance (Dredge et al. 2013). However, the effect of aerosols is significant during speleothem hiatuses (slow growth) and high aerosol deposition, which is a result of efficient transport and higher concentrations of these fine particles. Also, these aerosol deposits may aid microbiological associations which may, through bio-accumulative processes, produce inorganic horizons, as some microorganisms are capable of concentrating metals (Dredge et al. 2013 and the references therein). Historically, PM pollution in the cave was largely associated with soot emitted from the first tourist visitors using torches and other open-fire light sources (Gams 2004; Zupančič et al. 2011; Šebela et al. 2015) as well as emissions from natural and anthropogenic external sources, such as large-scale manmade or natural fires (e.g. Šebela et al. 2015, 2017), and re-suspended dust from regional (Christoforou et al. 1994) and distant dust storms, i.e. Sahara, East and Central Asia (Moulin et al. 1997; Frumkin and Stein 2004; Bou Karam et al. 2010; Formenti, et al. 2010; Millán-Martínez et al. 2021; Francis et al. 2022; Tositti et al. 2022). After the Industrial Revolution in particular, the sources of PM changed and have been predominantly related to human activities, such as coal-based industries (Christoforou et al. 1994; Salmon et al. 1994; Liu et al. 2011), traffic and emissions from the burning of biomass (e.g. Jeong et al. 2003; Chang et al. 2008). With the rise of modern cave tourism (Tičar et al. 2018), particularly uncontrolled mass tourism, the situation worsened (Michie 1999; Kurniawan et al. 2017).

In the past, some microclimatic cave studies were conducted in Škocjan Caves in Slovenia (Vercelli 1931; Kranjc and Opara 2002; Grgić et al.^2014^; Mulec et al. 2017; Debevec et al. 2018; Debevec and Rakovec 2021). Only a few regular measurements of particulate matter have been performed in the Škocjan (Grgić et al. 2014; Debevec and Peric 2016) and Postojna (Iskra et al. 2010; Bezek et al. 2012) caves, but without any characterization or attribution of source of particulate matter. The same is true for some other European caves, for example Ingleborough cave in the UK (Smith et al. 2013), the Císařská and Sloup-Šošůvka caves in Czech Republic (Faimon et al. 2011, 2012, 2019) and the Safranbolu Bulak Mencilis cave in Turkey (Cetin et al. 2017). Licbinsky et al. (2020) tried to identify the source of PMs by establishing their chemical composition. As the same chemical elements could be bound to different solid carriers, their mineralogical characterization is crucial in identifying the source. Therefore, individual particle analysis could contribute to the general identification as well as specific identification of the sources and origin of recent PMs in show caves. Additionally, in recent years, particular attention has been paid to ensuring sustainable visits to the caves and providing educational and awareness-raising activities on the surface of the park (Debevec et al. 2018).

The aim of our research was to (a) determine the relationship between PM concentrations and the microclimatic parameters of natural and/or anthropogenic origin in the cave (i.e. air temperature, CO_2_, natural air circulation and ventilation caused by opening the doors between the passages, effect of the river in the cave) over two different seasons (winter and summer) along the tourist path; (b) characterize the PMs microchemically, micromorphologically and mineralogically and assess their sources and finally (c) determine the relation between the characteristics of the PM and the number of visitors and tourist visits.

The results provide new insights into the impact of tourism on karst show caves by employing a novel integrated methodological approach, which enables us to establish the causal relationship between all of the factors involved. The findings of our study will help cave management to undertake appropriate measures to upgrade sustainable use of the UNESCO-listed cave for tourism.

## Material and methods

### Site description

The area of the Škocjan Caves (Slovenia) was designated as a UNESCO World Heritage Site in 1986 (Debevec Gerjevič and Jovanovič 2006) and also listed on the Ramsar List of Wetlands of International Importance in 1999. The cave has 6138 m of known passages and is 254 m deep (Cave Registry of the Republic of Slovenia 2022). In the geological sense, the passages of the Škocjan Caves are developed within a 300-m-thick sequence of Cretaceous (older Sežana Formation and younger Lipica Formation) and Cretaceous-Paleocene limestones (Liburnian Formation) (Šebela and Novak 2023). Strata in the Škocjan Caves area have a Dinaric strike orientation trending NW–SE and generally dipping towards the SW. Tiha Jama, where PM levels have been measured, is developed in a Liburnian Formation (K-Pc), comprising bedded limestones (containing the foraminifera genus *Alveolinae*). The area is cut by Dinaric-oriented (NW–SE) faults that may still be active (Šebela and Novak 2023). The reka River runs on the non-carbonate Eocene Flysch rocks (Jurkovšek 2013) and sinks into the underground of Škocjan Caves from the Eastern side.

**Fig. 1:**
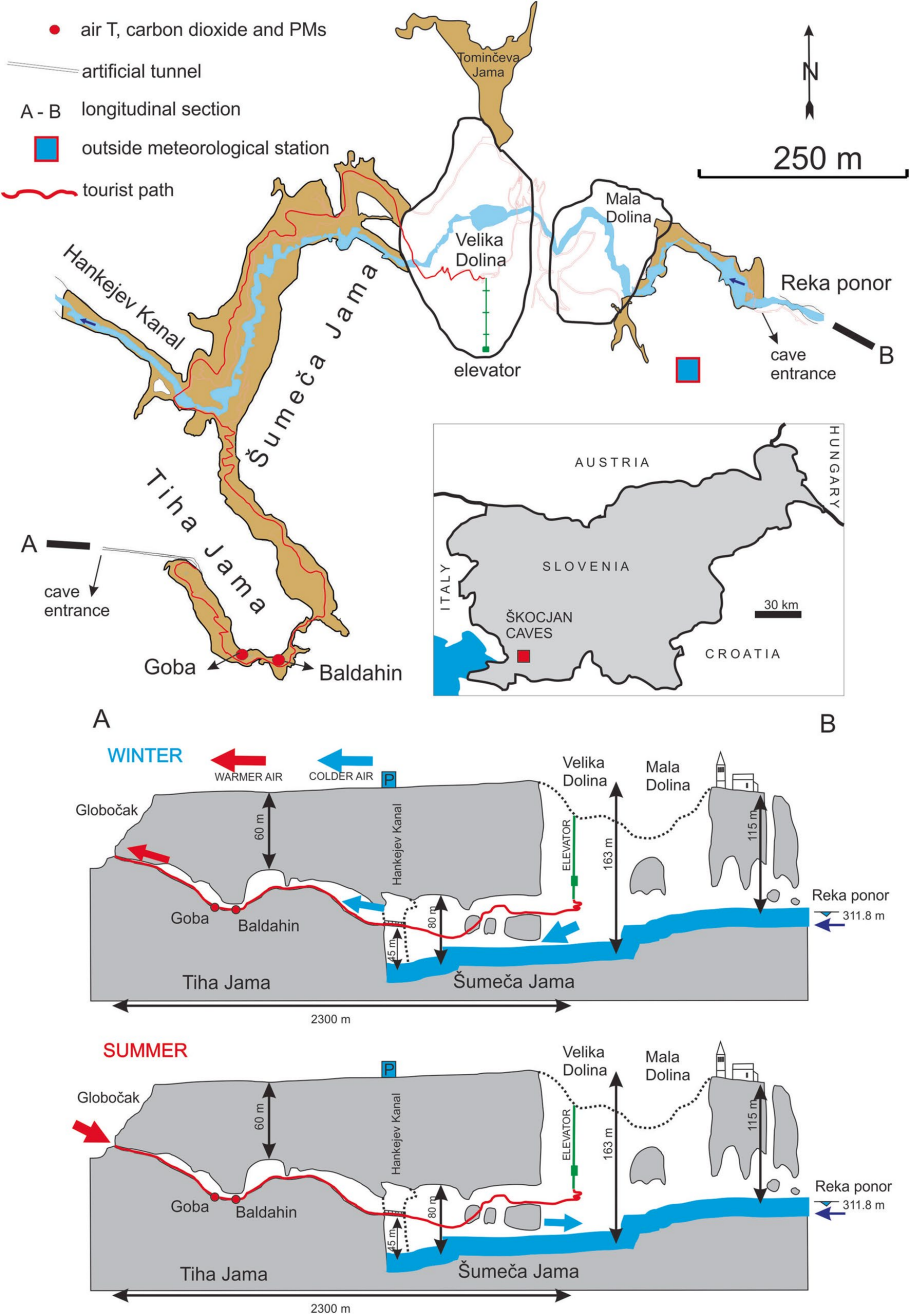
Schematic map of the Škocjan Caves with measurement and sampling locations (air T, CO_2_, PM, cave sediment) and schematic profile (A, B) of winter and summer ventilation modes (modified from Debevec and Rakovec 2021)

The Škocjan Caves are composed of two principal parts: the underground canyon of the Reka River passage that is up to 158 m deep and dry passages above it (Tiha Jama with Baldahin and Goba sites) that are connected to the surface via an artificial tunnel (Fig. 1) with doors that open for the visitors’ entrance. The Škocjan Caves have at least two openings at different levels (Fig. 1). Such caves represent an unconfined state in both winter and summer due to a chimney effect (Bourges et al. 2006). In winter (Fig. 1), when the outside temperature is lower than the temperature in the cave, cold air enters the cave through the lower opening (ponor or sinking point of the Reka River into the cave), which then becomes heated inside the cave and escapes via the upper (artificial tunnel) opening (Debevec and Rakovec 2021). In summer, when outside temperatures are higher than the temperature in the cave, warmer outside air is dragged into the cave through the higher entrances and cooler cave air escapes from the cave via the lower entrance (Fig. 1). Tiha Jama is characterized by stable inner cave climate parameters, especially air temperature, which is close to the mean annual surface temperature (Šebela 2022), while CO_2_ concentrations are higher due to natural conditions (Goba site) and decrease after the door opening in the tunnel (Debevec and Rakovec 2021). Also, along the Reka River cave passage, Prelovšek et al. (2021) observed a higher impact of external conditions on the cave microclimate.

PM, CO_2_ and air temperature were measured at two locations, Goba and Baldahin, in Tiha Jama (Fig. 1). The sampling sites were in a dry part of the cave, out of the reach of underground river floods and are situated 60 m above the bottom level of the river canyon and 500 m from it (Fig. 1). Both sites are represented by piles of cave sediments some 1–2 m high and some 2–3 m from the tourist path and some 50 m from each other. The Goba monitoring location is situated at 315 m and Baldahin at 317 m above sea level. The cave passage between the Goba and Baldahin sites is up to 10 m high and up to 20 m wide (Fig. 1). High flooding of the underground Reka River in Šumeča Jama can reach 307.5 m above sea level, as was the case in February 2019 (Blatnik et al. 2020).

The first documented visits into the Škocjan Caves took place as early as 1750 (Kranjc and Opara 2002). At the time of our research, a maximum of 400 tourists per day during winter and up to 2400 tourists per day during the summer were recorded (Prelovšek et al. 2021). The cave is open for visits at 10:00, 13:00 and 15:00 in winter, when the number of people per group is less than 20. In the summer, visits are organized every hour from 10:00 to 17:00, with additional groups also scheduled in-between. During our field research, group sizes were seldom below 50 people, even climbing to more than 250 persons (number of visitors hourly for 2018 according to Škocjan Caves Park management).

### Measurements and sampling of cave microclimatic parameters

The 7-day measurement and sampling campaigns of microclimatic parameters (air temperature (*T* in °C, CO_2_ in ppm) and cave particulate matter (PM in µg/m^3^) were performed at each location (Fig. 1) during both summer and winter. At both locations, instruments were placed about 2 to 3 m from the tourist path and about 0.5 to 1 m above the cave floor. For field measurements, we selected two locations in Tiha Jama (Fig. 1) as we wanted to determine whether small-scale heterogeneity (50 m distance) produced any differences in cave microclimate and PM characteristics. At the Baldahin site, measurements were carried out from 29 January to 5 February 2018 and from 30 August to 6 September 2018. At the Goba site, measurements were taken from 5 to 12 February 2018 and from 6 to 13 September 2018. Air *T* was measured at 5-min intervals with Eijkelkamp baro-divers (accuracy ± 0.1 °C, resolution ± 0.01 °C). CO_2_ volume concentrations in cave air were measured at 30-min intervals using a Vaisala GM70 CO_2_ meter (accuracy ± (1.5% of range + 2% of reading), resolution 10 ppm). Relative humidity (RH) values in Slovenian caves are generally very close to 100%. Commonly used sensors are not suitable for constant measurements under these conditions; therefore, RH measurements in the caves are not recommended (Mlakar et al. 2020).

### Measurements and sampling of cave PM and cave sediment

Cave PMs with aerodynamic diameters of less than 10 µm, less than 2.5 µm and less than 1 µm, together with their mass concentrations (PM_10_, PM_2.5_ and PM_1_, respectively), were measured and sampled using a portable laser aerosol spectrometer (OPC) Grimm 1.109. Data output (storage interval) was set at 5-min intervals. The gravimetric factor (C-factor) was arbitrarily set to 1. Sample airflow was 1.2 l/min. The filtered air volume ranged between 12 and 15.3 m^3^. All airborne PM was collected on original Grimm polytetrafluoroethylene (PTFE) membrane filters with a diameter of 47 mm. The OPC is calibrated at the producer (Grimm Aerosol Technik) every 6 months for particle size measurement using NIST-certified mono disperse polystyrene latex, and for the dust mass calculation using poly disperse dolomite dust compared to a reference device.

At both measuring sites, the upper 3 mm of the cave sediment was collected from the surface in order to better define the origin and possible sources of cave PM.

From the obtained data, we calculated the descriptive statistics, correlations, *t* tests and time series analyses (Single spectrum Fourier analysis; Swan and Sandilands 1995; Bourges et al. 2006) with TIBCO Statistica13.3 software (TIBCO 2017). As time series analyses require the same time interval between observations, we estimated CO_2_ values by interpolating two adjacent 30-min measurement points. For time series analyses, we considered data on the number of visitors at the approximate time they entered the cave and approached the sampling location, where they presumably stayed for 15 min.

### Characterization of cave PM and cave sediment

For individual particle analysis, the OPC filter and dried cave sediment were coated with a thin layer (10 nm) of carbon for conductivity. All samples were analysed and observed in backscattered electron (BSE) and secondary electron (SE) modes in a high vacuum using a scanning electron microscope (SEM) JEOL JSM 6490LV coupled with an Oxford INCA Energy 350 energy-dispersive spectroscopy (EDS) system at accelerating voltages of 5 to 20 kV; spot sizes of 20, 28 and 50 and a working distance of 10 mm. Some 21 to 29 representative fields of view were selected in each sample, in which individual particles were characterized according to size, morphology and chemical composition measured by using semi-quantitative EDS point analysis with an acquisition time of 60 s. Particle size was measured using a tool included in the JEOL SEM software (JEOL 2007). Possible mineral equivalents were assessed by comparing the atomic proportions of the constituent elements, excluding carbon and fluorine that are present in the PTFE filter, with the atomic proportions of the constituent elements in known stoichiometric minerals as obtained from mineral databases (Anthony et al. 2009; Barthelmy 2010), according to established procedure (Miler and Mirtič 2013).

The relative mineral and phase abundances in the cave sediments were estimated from the distribution of constituent elements obtained by the EDS elemental mapping of a randomly selected field of view in each sample at magnifications of × 300 to × 450 with an acquisition time of 700 s using the Area Measurements tool included in the INCA Energy software (Oxford Instruments 2006). The software was calibrated for quantification every 2 h using premeasured universal standards included in the EDS software, according to fitted standards procedure (Goldstein et al. 2003) and referenced to a co-optimization standard. Correction of the EDS data was performed on the basis of the standard ZAF-correction procedure included in the INCA Energy software (Oxford Instruments 2006).

Mineral composition of cave sediment samples was also determined by X-ray powder diffraction (XRD) using a Philips PW 3830/40 diffractometer with CuKα radiation at a voltage of 40 kV, a current of 30 mA and a graphite monochromator. Data was recorded in the range of 2° ≤ 2θ ≤ 70°. The results were obtained using PANalytical X’Pert Highscore Plus software. The content of the silt fraction of the cave sediment was determined using a Fritsch Analysette 22 NanoTec.

## Results and discussion

### Influence of visitors on cave microclimatic parameters and PM concentration

**Fig. 2:**
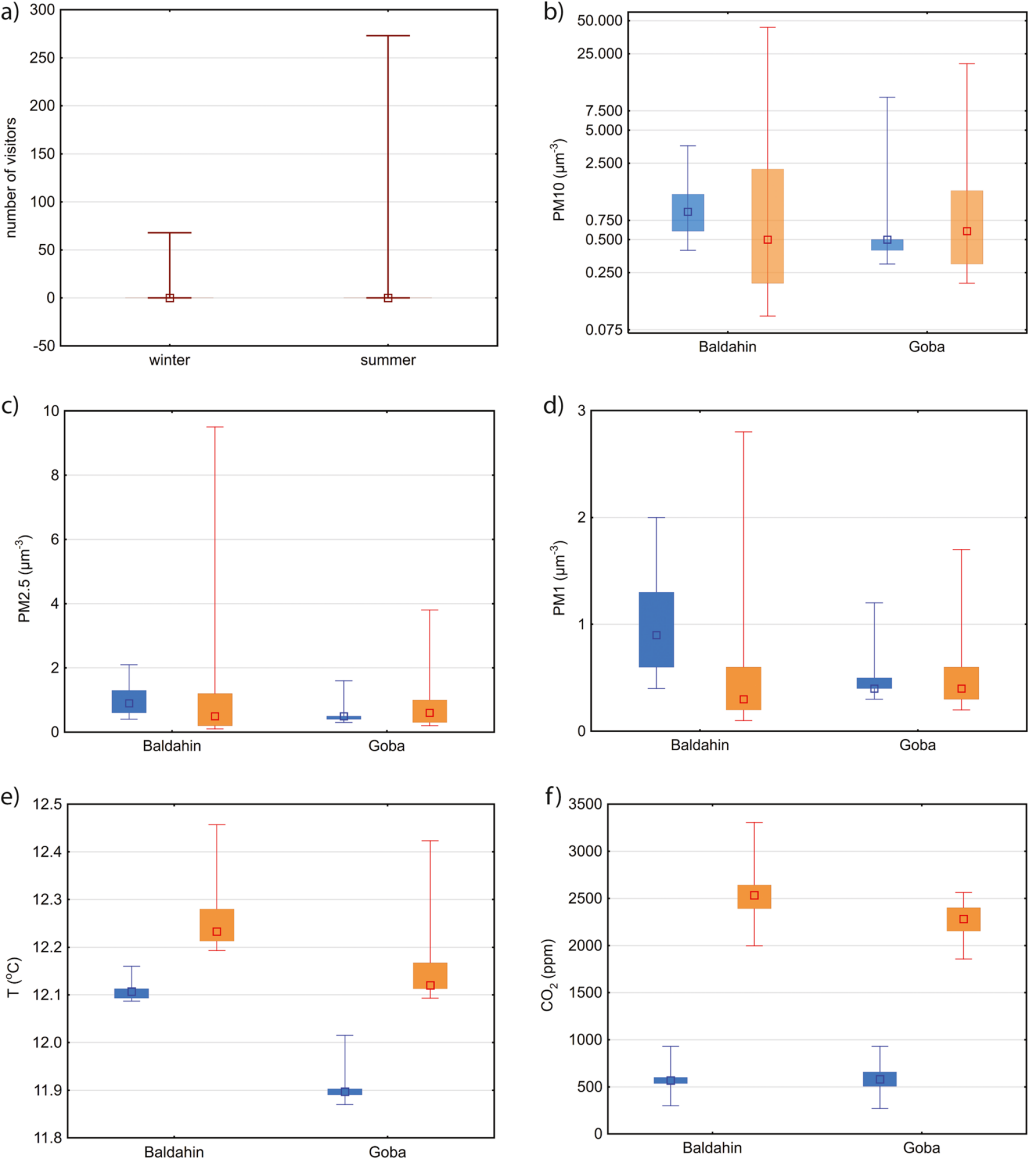
Box-whisker diagrams comparing **a** number of visitors, **b**, **d** PM concentrations and **e**, **f** microclimatic parameters in winter (blue) and summer (orange) at both locations. Legend: middle-point—median, box—interquartile range, whisker—min–max range

Statistical Student’s *t* tests show that measured variables (*T*, CO_2_ and PM fractions) vary by season and location. For most variables (Table S1; Fig. 2), larger mean and maximum values are observed in summer, when the number of visits and visitors is higher. However, the median and minimum values of PMs are always higher in winter (Fig. 2b–d). The correlation coefficients (Table S2) are above 0.9 and statistically significant only between all three PMs in winter at Baldahin. In summer, the correlations between PMs at Baldahin are lower and are influenced by some outliers. In the case of Goba, the correlations in winter and summer are slightly lower than at Baldahin, and between PM_10_ and PM_1_, in summer only 0.44 and in winter 0.26. In general, the observed mean values for all variables are higher at Baldahin than at Goba. Much higher visitor numbers and CO_2_ concentrations in summer versus winter were observed (Fig. 2a, f). At Baldahin, the average *T* is 0.25 °C higher in winter and 0.1 °C higher in summer than at Goba (Fig. 2e). The reason must lie in local microclimatic differences that are probably related to different cave morphologies and due to “unknown” inflow of cooler air from the limestone rock massif behind the Goba site (from fractures with percolating colder water). In summer, the CO_2_ concentration is on average 270 ppm higher at Baldahin than at Goba (Fig. 2f). At both sites, PM_10_ concentrations are on average more than twice as high in summer than in winter, the reason for which is the specific cave environment and position of the cave. The cave is located in a sparsely populated area, visited mainly by tourists in summer. Denser traffic in the cave area and on the nearby highway combined with more visits and visitors in the cave are the reason behind the higher PM levels in summer. Mean PM_2.5_ concentrations are similar at Baldahin in both seasons, but at Goba, they are 1.6 times higher in summer. On average, PM_1_ concentrations are half as low in summer than they are in winter, due mainly to their higher winter concentrations at Baldahin, indicating their different or additional sources. Baldahin is located closer to the Reka River canyon, and the elevated PM_1_ concentrations could be influenced by river spray. Increased aerosol production by turbulent water flow has already been reported for the Inglebourough show cave in the UK (Smith et al. 2013). However, the median PM_1_ levels are quite similar; only at Baldahin, they are almost twice as high in winter, and the median PM_1_ concentration is always slightly lower than that of the other two fractions. This is partly in agreement with the findings of Grgić et al. (2014) and Debevec and Peric (2016), who measured PM_10_ and nanoparticles in different seasons of the year and observed the highest aerosol concentrations in the summer and the lowest in the winter, when the visitor numbers were low. They associated the higher concentrations with the higher number of visitors and the infiltration of polluted external air due to door openings. They also found that during the summer, PM_10_ concentrations outside the cave are two to four times higher than inside the cave (Grgić et al. 2014). The same effect of visitors on PM concentrations was also confirmed in some other research (e.g. Smith et al. 2013; Cetin et al. 2017; Licbinsky et al. 2020). The observed PM concentrations in the cave are low, yet their compositions and sources can be considerably problematic. Even at low concentrations, PMs can affect the aesthetic value of the cave, as in the case of blackened speleothems (e.g. Liu et al. 2011; Dredge et al. 2013). Therefore, we support the sustainable use of the cave by controlling the daily number of visitors, already recently set in action. At present, a maximum of 1200 visitors can enter the cave over a single day, and visitor groups cannot exceed 150 people (personal communication, 2024).

**Fig. 3:**
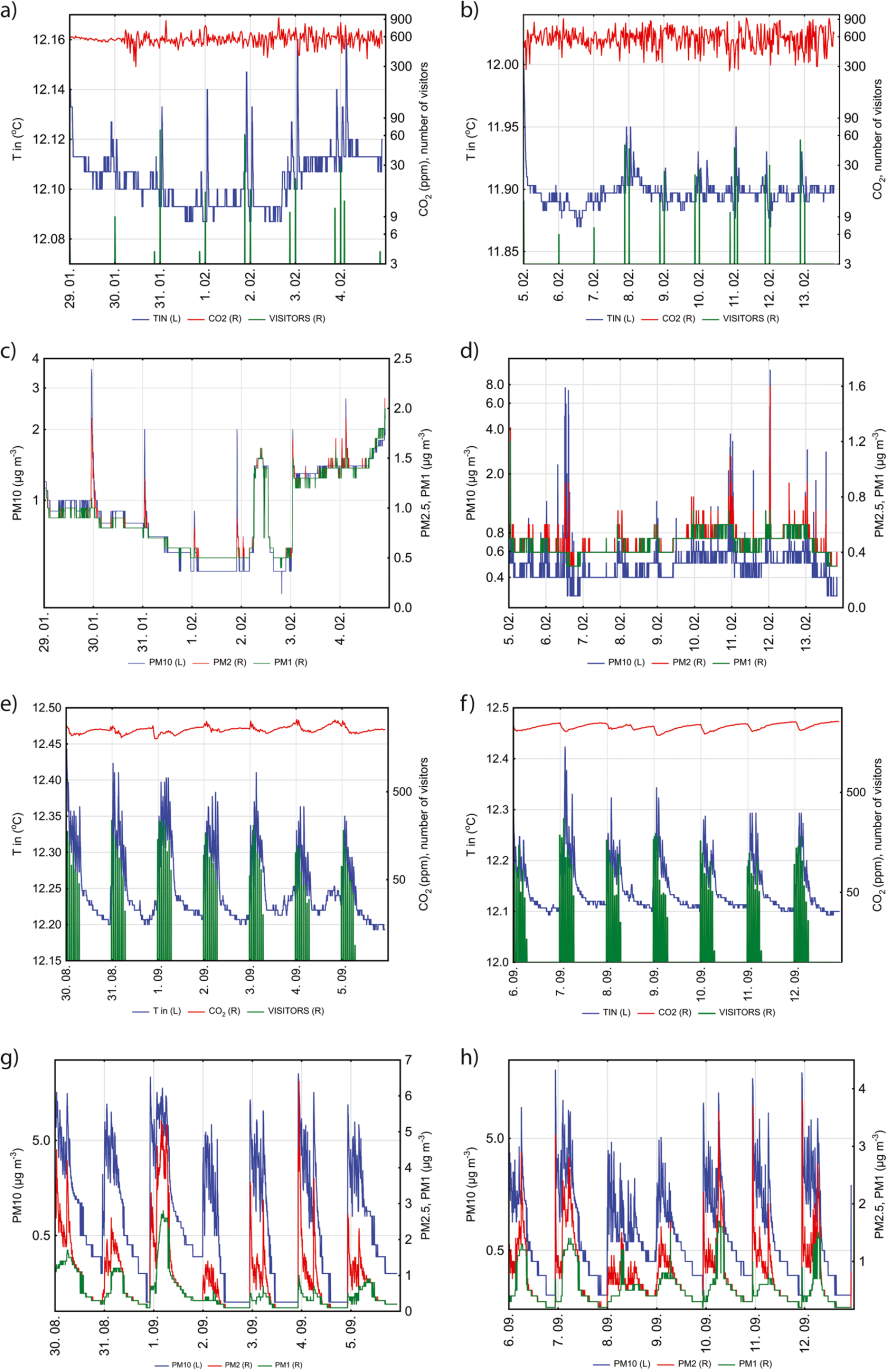
Time series of **a** cave temperature (*T* in °C; left *y*-axis), CO_2_ (ppm) and number of visitors (right *y*-axis) at Baldahin; **b** cave temperature (*T* in °C; left *y*-axis), CO_2_ (ppm) and number of visitors (right *y*-axis) at Goba; **c** PMs (in µg/m^3^) at Baldahin; **d** PMs (in µg/m^3^) at Goba; measuring locations in (**a**) to (**d**) were Baldahin from January 29 to February 5, and Goba from February 5 to February 13; **e** cave temperature (*T* in °C; left *y*-axis), CO_2_ (ppm) and number of visitors (right *y*-axis) at Baldahin; **f** cave temperature (*T* in °C; left *y*-axis), CO_2_ (ppm) and number of visitors (right *y*-axis) at GobaY; **g** at Baldahin; **h** PMs (in µg/m.^3^) at Goba. Measuring locations in (**e**) and (**h**) were Baldahin from August 30 to September 5 and Goba from September 6

Examination of the time series (Fig. 3) shows that the patterns are much more pronounced in summer than in winter. There is a relationship between cave visits and PM, *T* and CO_2_ changes in summer. The influence of cave visits on PM and *T* in winter is apparent but less pronounced. This is due to the lower number of visits and visitors. Normally, after opening the cave door and entering the cave, there is a sharp increase in temperature and all PM concentrations and a decrease in CO_2_ concentrations, followed by a slow decrease/increase until the next visit. Human activity clearly has an influence on the microclimatic parameters and PMs, as was already established by several authors (Smith et al. 2013; Cetin et al. 2017; Licbinsky et al. 2020). Because PM_10_ are larger, their concentrations decrease more rapidly than concentrations of the two smaller fractions, which is more evident in summer (compare Fig. 3b, d). It also appears that the peak concentrations of PM_1_ are somewhat delayed compared to those of the PM_10_, which is also reflected in a weaker correlation between the two.

In order to confirm possible multiple cyclicity in the data, we performed a time series analysis using single-spectrum Fourier analysis. Due to the observed seasonal and spatial differences, the analyses were performed separately for Baldahin and Goba in winter and summer. For all variables, the diurnal cycle is most pronounced in summer at both sites (Figs. S1 – S4). It is less pronounced in winter and shifted to a slightly shorter period of about 19 h for PM and in some cases for CO_2_ (21 h) in the case of Baldahin. For most variables, the closest recognized period is a half-day cycle. Exceptions are *T* in winter and PM_10_ and PM_2.5_ in summer at Goba, where a slightly shorter cycle of 8 h was observed. The 8-h cycle is also observed for PM_1_ and CO_2_ in summer at Goba but is less pronounced. All three cycles can be explained by the effect of opening the cave doors and differences in day versus night temperatures and ventilation on the cave microclimate and PM concentrations. The reason for less pronounced cycles in winter compared to summer is the smaller number of visits in winter. Also distinct is the 55-h cycle (2.3 days) at Baldahin for all PMs in summer and for CO_2_ in both seasons. It appears that some other as-yet unidentified natural phenomena influence the concentrations. For visitors only, a 1-h cycle with the second highest spectral density is observed in both seasons. The reason this cycle is missing for other variables could be due to the speed of changes and the influences of other climatic factors in the cave. Smith et al. (2013) reports that a period of 2 h after the last group of visitors is required for the cave atmosphere to recover.

### Granulometric analysis and relative mineral and phase abundance in cave sediment

Granulometric analysis of the cave sediment from both locations showed that 70–80% belongs to the silt fraction, and the rest is approximately equal proportions of sand and clay. Relative mineral and phase abundance as estimated based on EDS elemental mapping showed that cave sediments are predominantly composed of silicates, 72% at Baldahin and 51% at Goba, particularly K-aluminosilicates (41 to 44%), quartz (7 to 20%) and Na-aluminosilicates (2 to 7%). Carbonates are also abundant, with 25% at Baldahin and 46% at Goba, especially calcite and aragonite and minor dolomite. Present at both locations are also Fe-oxyhydroxides (1 to 1.5%), Mn oxides (0.4 to 0.6%) and Ti-bearing minerals and phases (0.4 to 0.8%).

### Characteristics and sources of individual particles in cave sediment

**Fig. 4:**
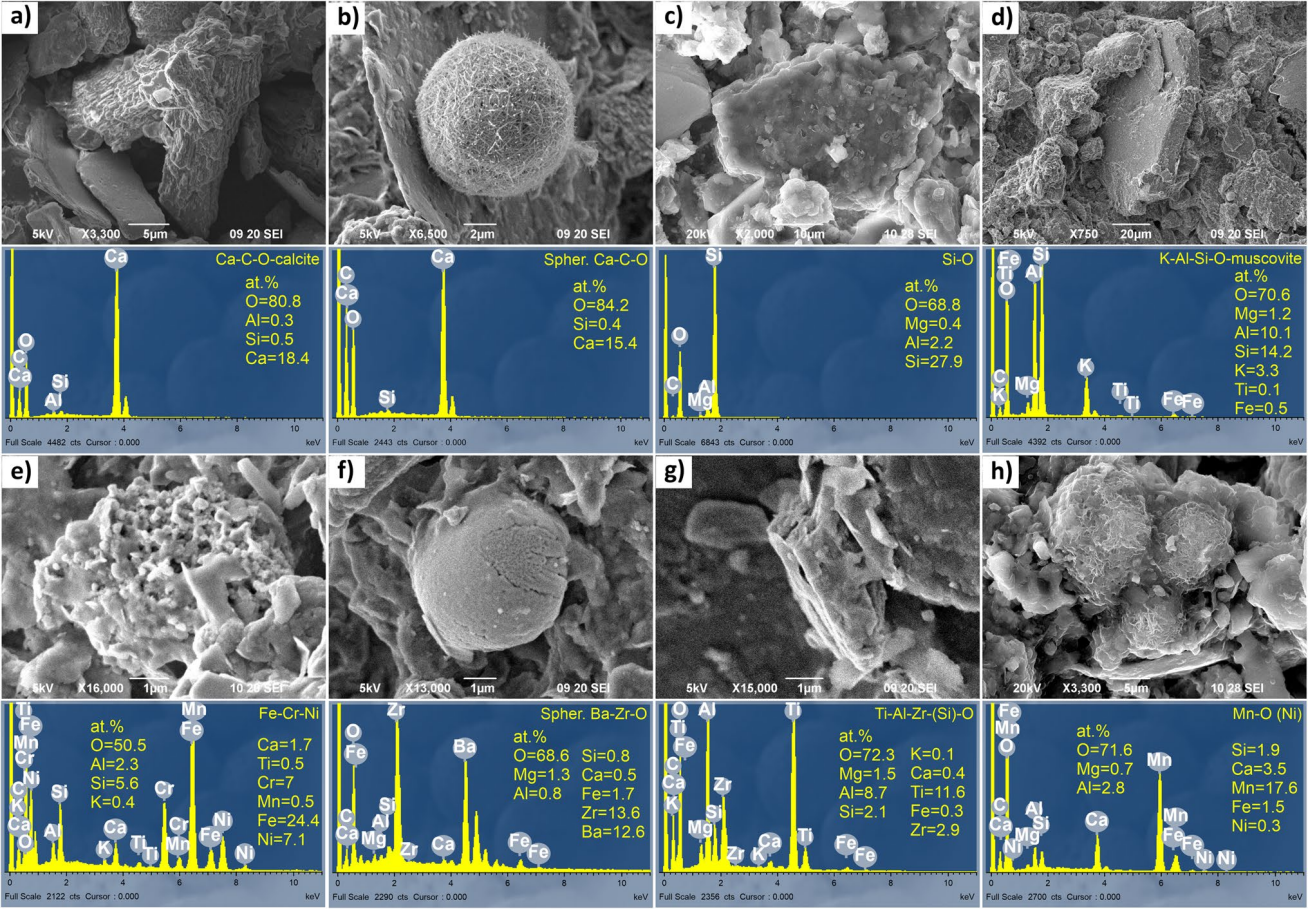
SEM (BSE) images, EDS spectra and semi-quantitative elemental composition of phases in cave sediment: **a** calcite (Goba); **b** spherical calcite (Baldahin); **c** quartz (Baldahin); **d** muscovite (Baldahin); **e** Fe–Cr–Ni alloy (Goba); **f** spherical Ba–Zr oxide (Baldahin); **g** Ti–Al–Zr–(Si) oxide (Baldahin); **h** Mn-oxide (Baldahin). The samples are coated with carbon

Identified phases in the cave sediments, which were considered only as reference background geogenic material and a possible source of PM material, together with their number and grain size are presented in Table 1. Images of representative phases are shown in Figs. 4 and S5. Of the individual particles in the cave sediment, 47 particles were analysed, and 21 different phases were identified (Table 1), which were classified as geogenic, anthropogenic and of mixed origin.

**Table 1:** Number (*n*), mean size (*d*_avg_) and standard deviations (std.) in μm of identified phases, their possible mineral equivalents and origin in cave sediment from the Baldahin and Goba locations

Origin	Phase	Possible mineral equivalent	Baldahin			Goba		
			*n*	*d*_avg_	std	*n*	*d*_avg_	std
G	Spher. Ca–C–O	Calcite	9	9.5	0.9	–	–	–
G	Ca–C–O	Aragonite	4	16.3	6.1	–	–	–
G	Ca–C–O	Calcite	3	20.5	2.7	3	10.0	3.9
G	Ca–Mg–C–O	Dolomite	2	25.9	4.6	2	22.6	0.8
G	Si–O	Quartz	3	36.2	12.9	2	10.2	6.1
G	K–Al–Si–O	Orthoclase	1	39.7	n.d	–	–	–
G	Na–Al–Si–O	Albite	–	–	–	1	20.9	n.d
G	Fe–Mg–K–Al–Si–O	Stilpnomelane	1	44.1	n.d	–	–	–
G	K–Al–Si–O	Muscovite	1	111.1	n.d	–	–	–
G	K–Ca–Fe–Mg–Al–Si–O	Illite	–	–	–	1	25.6	n.d
G	Al–Si–O	Kaolinite	–	–	–	1	25.9	n.d
G	Y–P–O	Xenotime	1	2.9	n.d	–	–	–
G	Ti–O	Rutile	1	17.7	n.d	–	–	–
A	Fe–O		1	4.9	n.d	–	–	–
A	Fe–Cr–Ni		–	–	–	1	6.5	n.d
A	Spher. Ba–Zr–O		1	5.4	n.d	–	–	–
A	Ti–Al–Zr–(Si)–O		1	5.6	n.d	–	–	–
M	C-bearing		1	40.1	n.d	–	–	–
M	Fe–O–OH	Ferrihydrite/Goethite	3	13.7	12.4	1	5.5	n.d
M	Mn–Fe–O		–	–	–	1	14.6	n.d
M	Mn–O (Ni)		1	22.6	n.d	–	–	–
	Total *n*		34			13		

*n.d.* not determined

– not identified

G geogenic; B biogenic; A anthropogenic; M mixed

#### Geogenic phases in cave sediment

Geogenic phases are most abundant at both locations. They are represented by calcite (Fig. 4a), spherical calcite (Fig. 4b) dolomite, quartz (Fig. 4c), orthoclase, albite, stilpnomelane, muscovite (Fig. 4d), aragonite (Fig. S5a), illite, kaolinite, xenotime and rutile.

##### Carbonates

Angular subhedral to euhedral calcite and dolomite grains occasionally exhibit oriented structured surfaces indicating multiple step growth (calcite) and elongated corrosion pits (dolomite) and occur at both locations. Long acicular euhedral aragonite crystals and spherical calcite were found only at Baldahin.

##### Silicates

Angular anhedral quartz grains were present at both locations, while orthoclase and albite appeared at Baldahin and Goba, respectively. Subangular platy grains of phyllosilicates stilpnomelane and muscovite were found at Baldahin and illite and kaolinite at Goba. XRD analysis of sediment samples confirmed the above results and revealed additional phases: the presence of quartz, illite/muscovite, chlorite/vermiculite and kaolinite, which is in accordance with studies by Zupan Hajna (1995) and Zupan Hajna et al. (2017). The sizes of the quartz and feldspars are somewhat larger at Baldahin and smaller at Goba (Table 1).

##### Phosphates and oxides

Angular anhedral xenotime and rutile were found only at Baldahin.

Most carbonates and silicates in the investigated cave sediments are typical detrital cave sediment minerals originating from the weathering of carbonate cave walls (Zupan Hajna 2002) and flysch rocks outside the cave and in the Reka River catchment area (Zupan Hajna et al. 2017), which served as a source of sediment, but aragonite and spherical calcite are authigenic. Xenotime and rutile are also detrital from flysch weathering.

#### Anthropogenic phases in cave sediment

Anthropogenic phases include Fe–Cr–Ni alloy (Fig. 4e), Ba–Zr oxide (Fig. 4f) and Ti–Al–Zr–(Si) oxide (Fig. 4g) and Fe-oxide (Fig. S5b).

##### Metal-bearing alloys and metal-bearing oxides/oxyhydroxides

Corroded anhedral Fe–Cr–Ni alloy was found only at Goba, while lamellar and striated Fe-oxide shavings were found only at Baldahin. Fe-oxide shavings and Fe–Cr–Ni alloy most probably result from maintenance work, such as the renovation and reconstruction of metal objects (fences, the bridge over the canyon, electrical cabinets, infrastructural cables) in the cave. During our studies, such activities were not on-going, but cave infrastructure was frequently under reconstruction.

##### Complex metal-bearing compounds

Smooth spherical Ba–Zr oxide and angular anhedral Ti–Al–Zr–(Si) oxide were found only at Baldahin. Ba–Zr oxide and Ti–Al–Zr–(Si) oxide do not correspond to any known natural mineral. The spherical shape of Ba–Zr oxide indicates it has been exposed to melting at high temperatures. These phases are commonly used in semiconductors and high-temperature electroceramics (Rendtorff et al. 2016), light-emitting diodes (Ivaniuk et al. 2017; Zhao et al. 2010) or less frequently welding electrodes (The Free Library 2014); therefore, they could originate from electronic devices, cave lightning or cave maintenance.

#### Phases of mixed origin in cave sediment

Phases whose origin could be interpreted in more than one way were classified as phases of mixed origin. These phases include Mn–Fe oxide and Mn-oxide (Fig. 4h), C-bearing particles (Fig. S5c), ferrihydrite or goethite (Fig. S5d).

##### Metal-bearing oxides/oxyhydroxides and C-bearing phases

Anhedral Mn–Fe oxide agglomerate of flaky crystals was present at the Goba location, while spherical agglomerates of minute bladed crystals of Mn-oxide with minor Ni content and platy angular anhedral C-bearing particles were found only at Baldahin. Spherical agglomerates (framboids) of euhedral cubes and individual octahedral crystals of ferrihydrite or goethite are common at both locations and could be either authigenic or detrital. The morphology and occurrence of Mn-oxide with minor Ni and Mn–Fe oxide content indicate that they are authigenic phases that formed inside the cave sediment. The Ni in the Mn-oxide could be the result of emissions from maintenance work in the cave. C-bearing particles (with minor S) can be interpreted as charcoal remains, which is consistent with observations of Šebela et al. (2015).

### Characteristics and sources of individual particles in cave PM

**Fig. 5:**
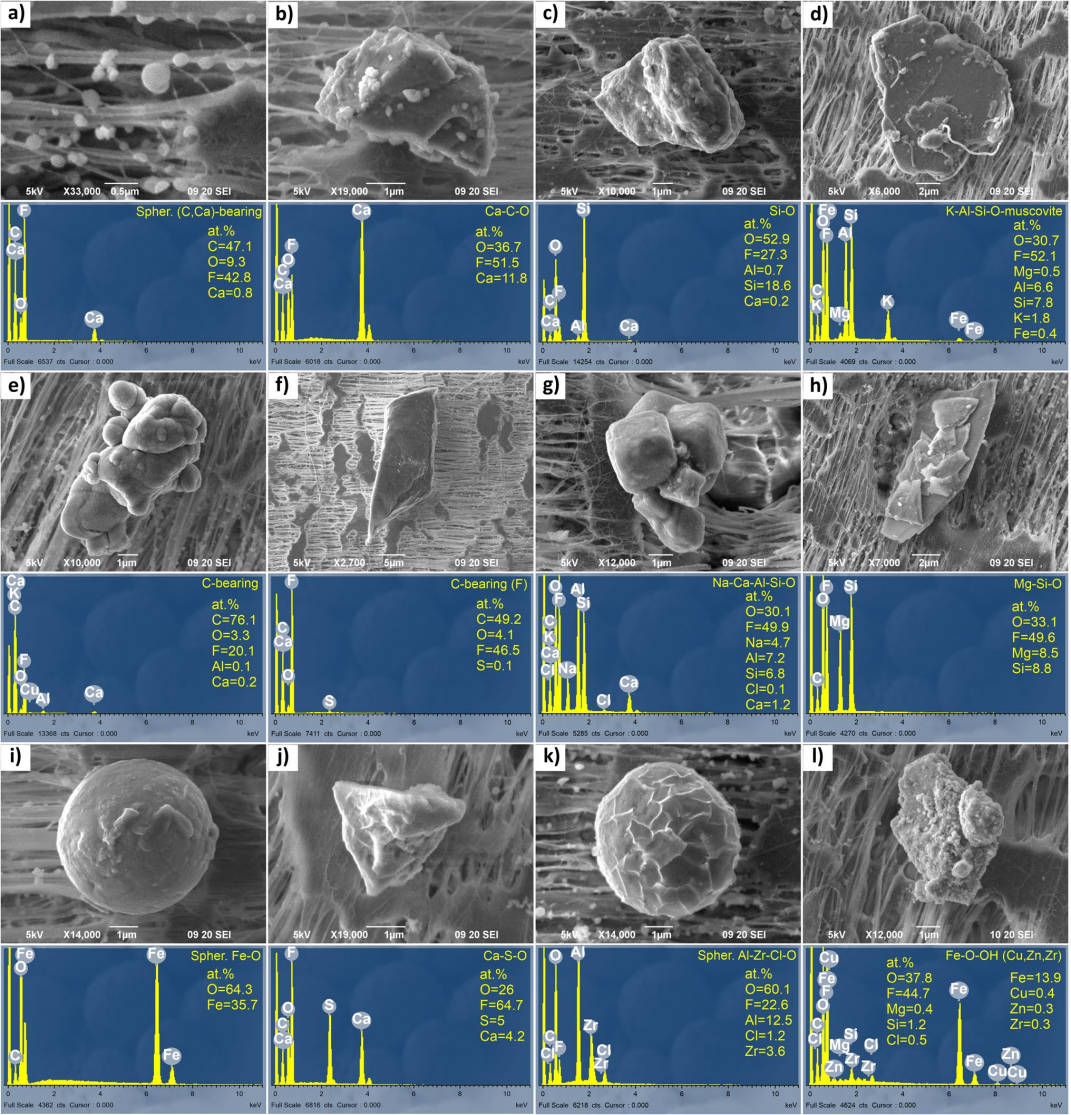
SEM (BSE) images, EDS spectra and semi-quantitative elemental composition of phases in cave PM: **a** spherical (C,Ca)-bearing particles (Baldahin, winter); **b** calcite (Goba, summer); **c** quartz (Baldahin, winter); **d** muscovite (Baldahin, summer); **e** pure C-bearing particle (Goba, winter); **f** C-bearing particle with minor F (Goba, summer); **g** natrolite or scapolite (Goba, summer); **h** Talc (Goba, winter); **i** spherical magnetite (Goba, summer); **j** gypsum or anhydrite (Baldahin, summer); **k** Spherical Al–Zr–Cl–O (Baldahin, winter); **l** ferrihydrite or goethite (Baldahin, summer). The samples are coated with carbon

Identified phases in the cave PM, together with their number and grain size, are presented in Table 2. Images of representative phases are shown in Figs. 5 and S5. The calculated weight of material collected on the OPC filter was between 6 and 7 µg in winter and between 16 and 27 µg in summer, which is relatively low. A total of 202 particles in the cave PM were analysed with 29 different phases identified (Table 2), which were classified as geogenic, anthropogenic, biogenic and of mixed origin.

**Table 2:** Number (*n*), mean size (*d*_avg_) and standard deviations (std.) in μm of identified phases, their possible mineral equivalents and origin on OPC filters from sampling locations at Baldahin and Goba sampled in winter and summer

Origin	Phase	Possible mineral equivalent	Baldahin			Baldahin			Goba			Goba		
			Winter			Summer			Winter			Summer		
			*n*	*d*_avg_	std	*n*	*d*_avg_	std	*n*	*d*_avg_	std	*n*	*d*_avg_	std
G	Spher. (C,Ca)-bearing		46	0.3	0.2	17	0.2	0.1	18	0.24	0.1	15	0.3	0.2
G	Ca–C–O	Calcite	12	3.4	3.9	12	7.3	3.8	6	4.0	1.1	7	5.6	2.3
G	Ca–Mg–C–O	Dolomite	1	5.5	n.d	–	–	–	–	–	–	1	5.7	n.d
G	Si–O	Quartz	2	8.5	1.6	1	3.6	n.d	1	7.7	n.d	3	5.3	1.3
G	Na–Al–Si–O	Albite	–	–	–	1	4.0	n.d	–	–	–	–	–	–
G	K–Al–Si–O	Muscovite	–	–	–	1	13.6	n.d	–	–	–	–	–	–
G	Fe–Mg–Al–Si–O	Chlorite	1	6.6	n.d	1	7.2	n.d	–	–	–	–	–	–
G	K–Ca–Fe–Mg–Al–Si–O	Illite	4	11.1	4.1	–	–	–	1	5.4	n.d	5	6.3	2.2
G	Agg. Si–O, Ca–Fe–Mg–K–Al–Si–O		–	–	–	2	6.3	4.2	–	–	–	–	–	–
G	Agg. K–Ca–Fe–Mg–Al–Si–O, Ca–C–O		3	4.4	4.2	2	8.6	1.7	2	6.0	2.3	–	–	–
G	Agg. Ca–C–O, Si–O		–	–	–	–	–	–	–	–	–	1	14.0	n.d
B	C-bearing (N,S)		–	–	–	–	–	–	–	–	–	1	19.6	n.d
A	C-bearing		1	4.0	n.d	1	4.9	n.d	4	7.7	3.1	–	–	–
A	C-bearing (F)		–	–	–	–	–	–	–	–	–	1	25.5	n.d
A	Na–Ca–Al–Si–O	Natrolite/Scapolite	–	–	–	–	–	–	1	5.9	n.d	1	6.6	n.d
A	Mg–Si–O	Talc	–	–	–	1	11.4	n.d	1	13.1	n.d	–	–	–
A	Na–Ca–Si–O		1	14.3	n.d	–	–	–	–	–	–	–	–	–
A	Spher. Ca–Mg–Fe–Na–K–Al–Si–O		–	–	–	1	2.6	n.d	–	–	–	–	–	–
A	Spher. Fe–O	Magnetite	–	–	–	–	–	–	–	–	–	3	2.2	2.7
A	Cu–O		–	–	–	–	–	–	1	5.6	n.d	–	–	–
A	Ca–S–O	Gypsum/anhydrite	–	–	–	1	3.9	n.d	–	–	–	–	–	–
A	Ba–S–O	Barite	–	–	–	1	0.6	n.d	1	2.2	n.d	–	–	–
A	Spher. Al–Zr–Cl–O		1	5.4	n.d	–	–	–	–	–	–	–	–	–
A	Agg. Na–Si–O, Zn–O		–	–	–	–	–	–	–	–	–	1	9.6	n.d
M	C-bearing with Ca–Al–Si–Fe–O		–	–	–	–	–	–	3	6.5	2.4	–	–	–
M	Fe–O–OH	Ferrihydrite/goethite	1	5.6	n.d	4	4.6	2.3	–	–	–	1	5.4	n.d
M	Agg. C-bearing, K–Ca–Fe–Mg–Al–Si–O, Ca–C–O		–	–	–	2	16.5	6.2	–	–	–	–	–	–
M	Agg. Ca–C–O, Ca–S–O, K–Ca–Fe–Mg–Al–Si–O, Fe–O–OH		–	–	–	–	–	–	–	–	–	1	15.3	n.d
M	Agg. Ca–C–O, K–Ca–Fe–Mg–Al–Si–O, Zn–Ba–S–O		–	–	–	1	4.9	n.d	–	–	–	–	–	–
	Total *n*		73			49			39			41		

*n.d.* not determined, – not identified

*G* geogenic, *B* biogenic, *A* anthropogenic, *M* mixed

#### Geogenic phases in cave PM

Geogenic phases are most abundant at both locations and seasons. They are largely represented by spherically shaped (C,Ca)-bearing particles (Fig. 5a), followed by calcite (Fig. 5b), quartz (Fig. 5c), albite, muscovite (Fig. 5d), chlorite, dolomite (Fig. S5e), illite (Fig. S5f), agglomerate of illite and calcite, agglomerate of quartz and Ca–Fe–Mg–K–Al–Si–O and agglomerate of calcite and quartz.

##### Spherical (C,Ca)-bearing particles

The largest number of very small spherical (C,Ca)-bearing particles was found at Baldahin in winter. We interpret these particles as amorphous calcium carbonate that precipitated in the cave due to very moist air saturated with dissolved calcium carbonate, assisted by bacteria action (Enyedi et al. 2020), and which are quite abundant and very diverse in the Škocjan Caves (Mulec et al. 2017), in moulds (e.g. Garvie et al. 2022) or in fungi (Bindschedler et al. 2016).

##### Carbonates

Calcite and dolomite are somewhat more abundant at Baldahin. Calcite may contain minor levels of Mg, Si and S. It occurs as anhedral angular grains, spherical and polycrystalline particles or single crystals (rhombohedrons, scalenohedrons) and as agglomerates with Al-silicates and metal-bearing oxides/oxyhydroxides.

##### Silicates

Platy (foliated) grains and agglomerates of illite and angular anhedral quartz are present at both locations. Angular anhedral albite, thin foliated muscovite with rounded edges and platy chlorite were found only at the Baldahin location.

Calcite, dolomite, quartz, albite and muscovite predominantly originate from the cave, as they are ubiquitous minerals in the Škocjan Caves and are also abundant in the investigated cave sediment. Carbonate grains in cave PM are far smaller than those in the cave sediment, indicating fine sediment resuspension as a consequence of moving cave air masses due to opening doors and visitors walking the tourist path. Chlorite and illite also result from cave sediment resuspension.

##### Complex agglomerates

Agglomerates of illite and calcite were found at both locations, while the association of quartz and Ca–Fe–Mg–K–Al–Si–O was present only at Baldahin in summer and the association of calcite and quartz occurred at Goba in summer. All these agglomerates are characterized by angular anhedral and anhedral platy grains. They are secondary particles that are most probably formed by the agglomeration of geogenic phases inside the cave.

#### Anthropogenic phases in cave PM

The second most abundant phases are anthropogenic phases. They are represented by pure C-bearing particles (Fig. 5e), C-bearing particle with minor F content (Fig. 5f), natrolite or scapolite (Fig. 5g), talc (Fig. 5h), spherical magnetite (Fig. 5i), gypsum or anhydrite (Fig. 5j), spherical Al–Zr–Cl–O (Fig. 5k), Na–Ca–Si–O (Fig. S5g), spherical Ca–Mg–Fe–Na–K–Al–Si–O (Fig. S5h), Cu-oxide (Fig. S5i), barite (Fig. S5j), agglomerate of Na–Si–O and Zn-oxide (Fig. S5k).

##### C-bearing phases

Pure C-bearing particles were found at both locations and have irregular shapes (summer samples) or occur as globular agglomerates or agglomerates of spherical particles (in winter samples). C-bearing particle with minor F has an irregular shape and is present only at the Goba location. The morphologies and chemical composition of these C-bearing particles are very similar to different types of microplastic particles, i.e. polytetrafluoroethylene (PTFE) and polyethylene (PE) microbeads. The first was already reported in marine environments (Ding et al. 2019), and the second is used as an exfoliant in cosmetics (Napper et al. 2015), and similar microbeads have already been detected in sediments of other show caves (Balestra and Bellopede 2022). As Reka River water and sediments inside the Škocjan Caves are polluted with plastic waste material and microplastic particles (Valentić et al. 2022) and most C-bearing particles in the cave PM were found in winter samples during winter ventilation mode, aerosols from Reka River spray could be one of the major sources of C-bearing particles. Globular C-bearing particles could also be part of larger fibres (clothes) that partly disintegrated into smaller pieces. Considering only chemical composition of the C-bearing particles, one could interpret their origin in solid or liquid fuel combustion outside the cave. However, particle morphology is very different from that of particles from fuel combustion, which includes hollow spheres with large pores due to the expulsion of gases, or porous sponge-like soot particles (e.g. Miler and Gosar 2009, 2015; Miler 2021). C-bearing particles in the cave PM also have different surface morphology compared to those in the cave sediment, those found in the insoluble residue of black deposit crusts obtained from speleothems in Črna Jama (Postojna Cave) and a black substance found in the soil profile outside the cave (Šebela et al. 2017).

##### Silicates

Assemblages of natrolite or scapolite pseudocubes with partly rounded edges were present only at Goba. Thin foliated sharp-edged talc was found at both locations, while thin platy sharp-edged Na–Ca–Si–O (most probably Na–Ca silicate glass) with a smooth surface and spherical Ca–Mg–Fe–Na–K–Al–Si–O with a smooth surface were found only at Baldahin. Morphologies of natrolite or scapolite, talc, Na–Ca silicate glass and spherical Ca–Mg–Fe–Na–K–Al–Si–O grains indicate short transport. Their sources were most probably emissions from maintenance work and other research-related activities within the cave (Ščuka 2019).

##### Metal-bearing oxides/oxyhydroxides

Spherical magnetite contains minor Zn and was found only at Goba in the summer, while anhedral partly corroded flaky agglomerate (or shaving) of Cu-oxide was present at Goba in winter. Spherical magnetite is an airborne phase formed during the melting of iron or steel at very high temperatures, followed by rapid cooling in the air (Umbría et al. 2004; Tasić et al. 2006). Its small size indicates transport from remote external sources, such as iron- and steel-based metallurgical industry (Miler and Gosar 2015; Šebela et al. 2015; Teran et al. 2020; Gaberšek and Gosar 2021; Miler 2021). However, local sources, such as the cutting and grinding of metals (Lu et al. 1992) during maintenance work within the cave, cannot be ruled out. According to its morphology and chemical composition, Cu-oxide results from the oxidation of metallic copper, which is common in wiring and electrical equipment and could originate from maintenance work in the cave.

##### Sulphates

Angular anhedral gypsum or anhydrite was found only in summer at Baldahin and angular to rounded anhedral barite at both locations—at Baldahin in summer and Goba in winter. Minor Sr content was detected in the barite. These phases have four possible sources. One of the sources of sulphate for the formation of gypsum or anhydrite could be a sulphidic spring in the dry riverbed 500 m upstream of the entry-point of the Reka River into the Škocjan Caves (Mulec et al. 2021). The sulphur in the spring probably originates in the nearby coal layers (Mulec et al. 2021). Maintenance work within the cave represents a second possible source, as these sulphates are basic, commonly used constituents of inorganic colouring pigments (Trimbacher and Neinavaie 2002) and mortars. A third source is airborne emissions from outside the cave, either from incomplete coal combustion and coal residue (Trimbacher and Neinavaie 2002; Wang et al. 2019) or from traffic emissions, since gypsum or anhydrite and barite are used as fillers in rubber and plastics (Harris 2003; Harben and Dickson 2006; Sharpe and Cork 2006) and in brake pads (Grigoratos and Martini 2015). The fourth possible source of gypsum and anhydrite is an industrial waste disposal site about 23 km southeast of the Škocjan Caves, which contains predominantly waste gypsum from acid production and where eroded waste material is washed into the Reka River (Černe Štemberger et al. 2018).

##### Complex metal-bearing compounds

Spherical Al–Zr–Cl–O with a plated, polygonal surface structure was found at Baldahin in winter. According to its composition and occurrence, it is most probably aluminium zirconium tetrachlorohydrex gly, a component of antiperspirants (Meyer 2019), introduced into the cave environment by visitors.

##### Complex agglomerates

An angular agglomerate of minute crystals of Na–Si–O and Zn–O occurred at Goba in summer. This phase is most probably Na-silicate glass with added Zn-oxide and could be part of water glass, which was probably used in maintenance work inside the cave.

#### Biogenic phases in cave PM

Phases of biogenic origin are represented only by irregularly shaped C-bearing particle with minor N and S content found at Goba in summer. Its surface morphology and composition indicate that it could be organic matter that formed within the cave or was part of some plant tissue.

#### Phases of mixed origin in cave PM

Phases of mixed origin are ferrihydrite or goethite (Fig. 5l), associations of C-bearing particles and Ca–Al–Si–Fe–O; agglomerate of C-bearing phases, illite and calcite; agglomerate of calcite, gypsum or anhydrite, illite and Fe-oxides/oxyhydroxides and agglomerate of calcite, illite and Zn–Ba–S–O (Fig. S5l).

##### Metal-bearing oxides/oxyhydroxides

Angular anhedral ferrihydrite or goethite, as well as minute crystals adhered to grain surfaces, is present at both locations, particularly in the summer samples. Most ferrihydrite or goethite grains contain minor Si and Al, while some also show traces of Mn, V, Pb, Cu, Zn, Zr and Mo, particularly those found in summer at Baldahin. This phase is a weathering product of either geogenic or anthropogenic Fe-bearing phases and an effective scavenger of various elements, particularly metals (Hudson-Edwards 2003; Gasparatos 2013) either of natural or anthropogenic origin. Their source could be the resuspension of cave sediment and the weathering of parent rock, as they occur in cave sediment and have already been found in parent rock of the same lithology and speleothems in nearby karst caves (Zupančič et al. 2016, 2018). Those with minor Pb, Cu, Zn and Zr are anthropogenic.

##### Complex agglomerates

Associations of C-bearing particles and Ca–Al–Si–Fe–O occur as globular agglomerates, which were found only at Goba in winter. Agglomerates of C-bearing phases, illite and calcite; agglomerate of calcite, gypsum or anhydrite, illite and Fe-oxides/oxyhydroxides and agglomerate of calcite, illite and Zn–Ba–S–O occur as angular anhedral agglomerates and anhedral platy agglomerates. Association of C-bearing phases (microplastics or coal residue), illite and calcite, including barite and minor S and Cr content, and association of calcite, illite and Zn–Ba–S–O (most probably white pigment lithopone) were found at Baldahin in summer. Agglomerate of calcite, gypsum or anhydrite, illite and Fe-oxides/oxyhydroxides was present at the Goba location in summer. All these agglomerates are associations of geogenic and anthropogenic phases that formed inside the cave; however, their constituents could be sourced from the cave sediment, from maintenance work in the cave and sources outside the cave as well.

### Grain size distribution of phases in cave PM

**Fig. 6:**
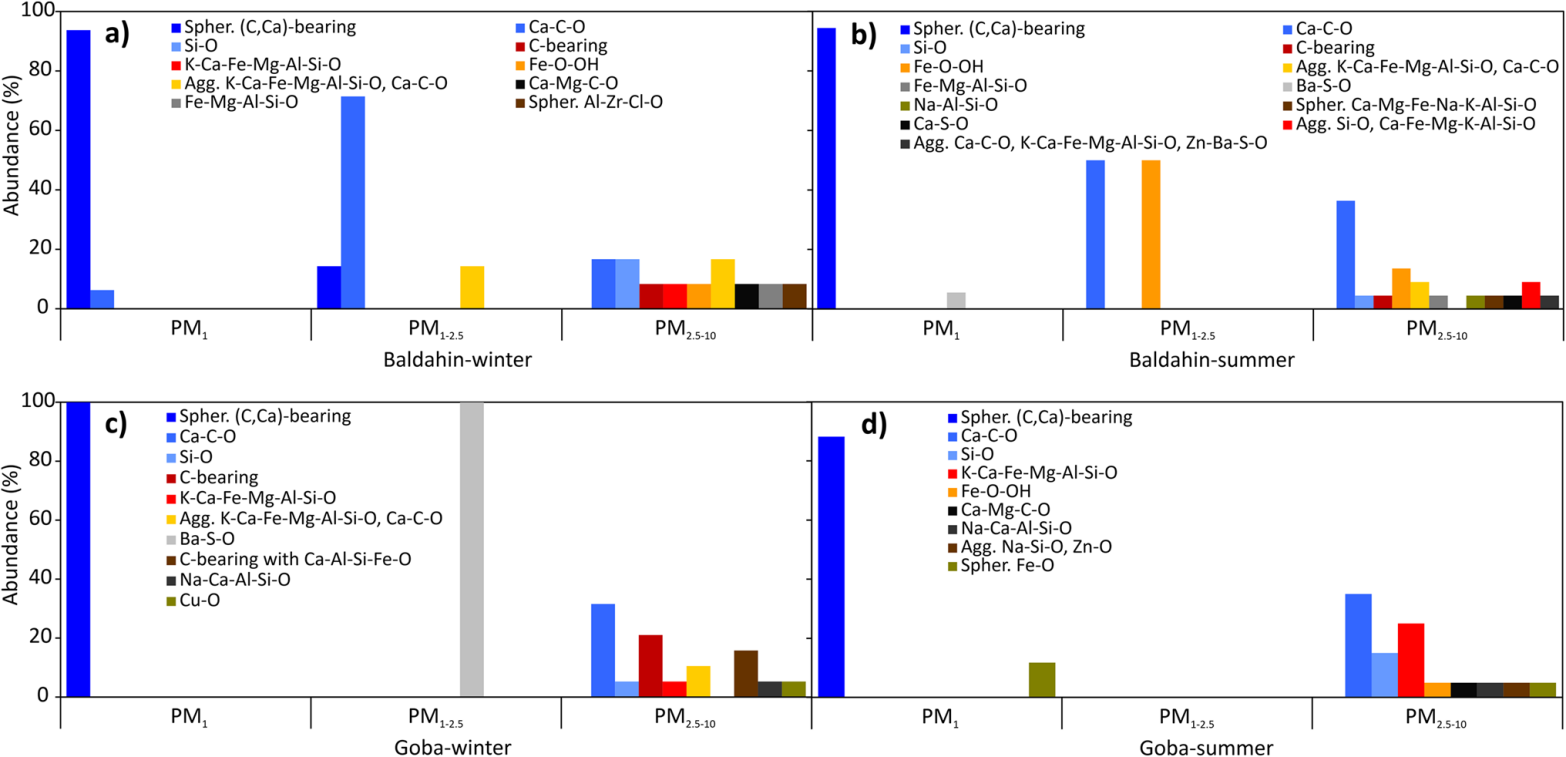
Grain size distribution of phases (in %) in cave PM from **a** Baldahin in winter; **b** Baldahin in summer; **c** Goba in winter and **d** Goba in summer among PM fractions

Grain size distribution of phases in cave PM at different locations and in different seasons is shown in Fig. 6 and is expressed as abundance (%) of phases in each PM fraction. Fraction PM_1_ (very fine) is predominantly represented at both locations in both seasons by spherical (C,Ca)-bearing particles (88–100%). At Baldahin, they are accompanied by calcite (6%) in winter and by barite (6%) in summer, while at Goba, they are accompanied by spherical magnetite (12%) in summer. Fraction PM_1-2.5_ (fine) consist of calcite (72%), spherical (C,Ca)-bearing particles (14%) and agglomerates of illite and calcite (14%) at Baldahin in winter. Calcite and ferrihydrite or goethite each represent 50% of fine fraction at Baldahin in summer. At Goba, fine fraction is represented only by barite in winter. Fraction PM_2.5–10_ (coarse) at both locations and seasons consists of most phases. At Baldahin in winter, coarse fraction is largely composed of calcite (17%), quartz (17%) and agglomerates of illite and calcite (17%), while other identified phases contribute each 8%. At Baldahin in summer, coarse fraction contains more calcite (36%), followed by ferrihydrite or goethite (14%), agglomerates of quartz and Ca–Fe–Mg–K–Al–Si–O (9%) and agglomerates of illite and calcite (9%). Other phases each contribute about 4%. Coarse fraction at the Goba site is dominated by calcite (32%), C-bearing particles (21%), agglomerates of C-bearing particles and Ca–Al–Si–Fe–O (16%) and agglomerates of illite and calcite (10%) in winter, while other phases are each represented by 5%. In summer, the coarse fraction is largely composed of calcite (35%), illite (25%) and quartz (15%); other phases each contribute 5%.

Grain size distribution helps us understand the sources of cave PM. The finest PM fraction seems most affected by the natural processes in the cave atmosphere, regardless of the season, namely the precipitation of amorphous calcium carbonate. Fine PM fraction is mostly fed from the resuspension of autochthonous cave sediment and the weathering of parent rock (carbonates) and maintenance work (metal-bearing oxides/oxyhydroxides, barite). The coarse PM fraction appears to be more dependent on the season and higher visitor numbers, resulting in the resuspension of autochthonous (carbonates) and allochthonous (silicates) cave sediment as well as anthropogenic material from past maintenance work.

## Conclusions

The measured microclimatic parameters and PM concentrations, with the exception of PM_1_, are higher in summer due to the far higher number of visits and visitors. They are also higher at Baldahin than at Goba, where the difference is the result of the cave morphology and the possible inflow of cooler air and percolating water from the fractures at Goba. The higher winter PM_1_ concentrations at Baldahin indicate that cave ventilation controls the introduction of very fine PM to the Škocjan Cave environment. The results of the time series analyses showed seasonal, semi-diurnal and 8-h cycles corresponding to the expected natural cycles and the opening of the cave doors, confirming that the number of visits and visitors has a strong influence on the parameters in summer. The opening of the cave doors leads to an immediate increase in cave temperature and PM and a decrease in CO_2_ concentration, followed by a gradual return to baseline values.

Most of the cave PM is represented by geogenic/biogenic minerals derived primarily from the resuspension of fine cave sediment by natural ventilation as well as by the opening of the cave doors to visitors. Phases of anthropogenic origin detected in the cave PM include emissions of microplastics from the polluted Reka River, maintenance activities in the cave and emissions from remote industry and traffic outside the cave. Other anthropogenic input pathways include direct input from visitors.

Grain size distribution of the phases showed that PM_1_ consists mainly of (C,Ca)-bearing particles, spherical magnetite and partially barite; PM_1–2.5_ consists mainly of calcite, ferrihydrite or goethite and barite, while all other phases fall into the PM_2.5–10_ fraction.

The study indicates that a novel methodological approach combining characterization and source apportionment of individual particles is particularly important for undertaking appropriate and effective measures to control PM input into the cave and thus contribute to maintaining a good cave environment.

Therefore, we propose control of cave maintenance activities and regular monitoring of PM in the cave, in addition to the existing monitoring of microclimatic parameters. Regular detailed identification and characterization of the individual particles in PM and their sources is also highly advisable.

### Supplementary Information

Below is the link to the electronic supplementary material.

Supplementary file1 (PDF 1884 KB)

## Data Availability

Data will be available upon request.
