# Supplementary material for: Natural and anthropogenic impact on the microclimate and particulate matter in the UNESCO show cave
Source: Environ Sci Pollut Res Int. 2024 Jul 19;31(35):48313–31. doi: 10.1007/s11356-024-34366-8 (PMC11297898; doi:10.1007/s11356-024-34366-8)
Supplement: Supplementary file 1 — Supplementary file1 (PDF 1884 KB) [file 11356_2024_34366_MOESM1_ESM.pdf]

Supplementary data

Reflection of tourism on the UNESCO show cave meteorology and particulate matter

Miloš Miler[a], Nina Zupančič[b,c], Stanka Šebela[d], Simona Jarc[b]

[a]Geological Survey of Slovenia, Dimičeva ulica 14, SI-1000 Ljubljana, Slovenia; e-mail: milos.miler@geo-zs.si

[b]University of Ljubljana, Faculty of Natural Sciences and Engineering, Department of Geology, Aškerčeva 12, SI-1000 Ljubljana, Slovenia; e-mails: nina.zupancic@ntf.uni-lj.si; simona.jarc@ntf.uni-lj.si

[c]ZRC SAZU, Ivan Rakovec Institute of Palaeontology, Novi trg 2, SI-1000 Ljubljana, Slovenia

[d]ZRC SAZU Karst Research Institute, Titov trg 2, SI-6230 Postojna, Slovenia; e-mail: sebela@zrc-sazu.si

Table S1. Descriptive statistics of PM (µg/m^3), T (ºC), CO_2 (ppm) and number of visitors for Baldahin and Goba locations in winter and summer sampling periods.

Variable		Winter		Summer	
		Baldahin	Goba	Baldahin	Goba
Mean	PM_{10}	0.95	0.52	2.00	1.21
	$PM_{2.5}$	0.95	0.47	0.93	0.74
	PM_1	0.93	0.45	0.51	0.52
	T	12.10	11.90	12.25	12.14
	CO_2	569.69	582.50	2540.34	2270.88
	visitors	0.42		8.68	
Median	PM_{10}	0.90	0.50	0.50	0.60
	$PM_{2.5}$	0.90	0.50	0.50	0.60
	PM_1	0.90	0.40	0.30	0.40
	T	12.11	11.90	12.23	12.12
	CO_2	566.67	580.00	2534.00	2283.00
	visitors	0.00		0.00	
Minimum	PM_{10}	0.40	0.30	0.10	0.20
	$PM_{2.5}$	0.40	0.30	0.10	0.20
	PM_1	0.40	0.30	0.10	0.20
	T	12.09	11.87	12.19	12.09
	CO_2	300.00	270.00	1997.00	1857.00
	visitors	0.00		0.00	
Maximum	PM_{10}	3.60	10.00	43.50	20.30
	$PM_{2.5}$	2.10	1.60	9.50	3.80
	PM_1	2.00	1.20	2.80	1.70
	T	12.16	12.02	12.46	12.42
	CO_2	930.00	930.00	3306.00	2563.00
	visitors	68.00		273.00	

Table S2. Correlation matrices of all variables calculated for Goba and Baldahin in winter and summer sampling periods. Bolded are statistically significant correlations at $\alpha = 0.05$.

Baldahin - winter (n = 1994)

Variable	PM_{10}	$PM_{2.5}$	PM_1	T
PM_{10}				
$PM_{2.5}$	**0.98**			
PM_1	**0.93**	**0.99**		
T	**0.59**	**0.59**	**0.55**	
CO_2	-0.03	-0.03	-0.03	-0.02

Goba - winter (n = 2554)

Variable	PM_{10}	$PM_{2.5}$	PM_1	T
PM_{10}				
$PM_{2.5}$	**0.61**			
PM_1	**0.26**	**0.85**		
T	**-0.13**	**0.08**	**0.15**	
CO_2	**-0.07**	**-0.10**	**-0.07**	**-0.07**

Baldahin - summer (n=1996)

Variable	PM_{10}	$PM_{2.5}$	PM_1	T
PM_{10}				
$PM_{2.5}$	**0.89**			
PM_1	**0.61**	**0.84**		
T	**0.64**	**0.68**	**0.55**	
CO_2	**-0.09**	**-0.26**	**-0.48**	**-0.07**

Goba - summer (n = 1997)

Variable	PM_{10}	$PM_{2.5}$	PM_1	T
PM_{10}				
$PM_{2.5}$	**0.83**			
PM_1	**0.44**	**0.82**		
T	**0.44**	**0.60**	**0.47**	
CO_2	**-0.25**	**-0.53**	**-0.60**	**-0.69**

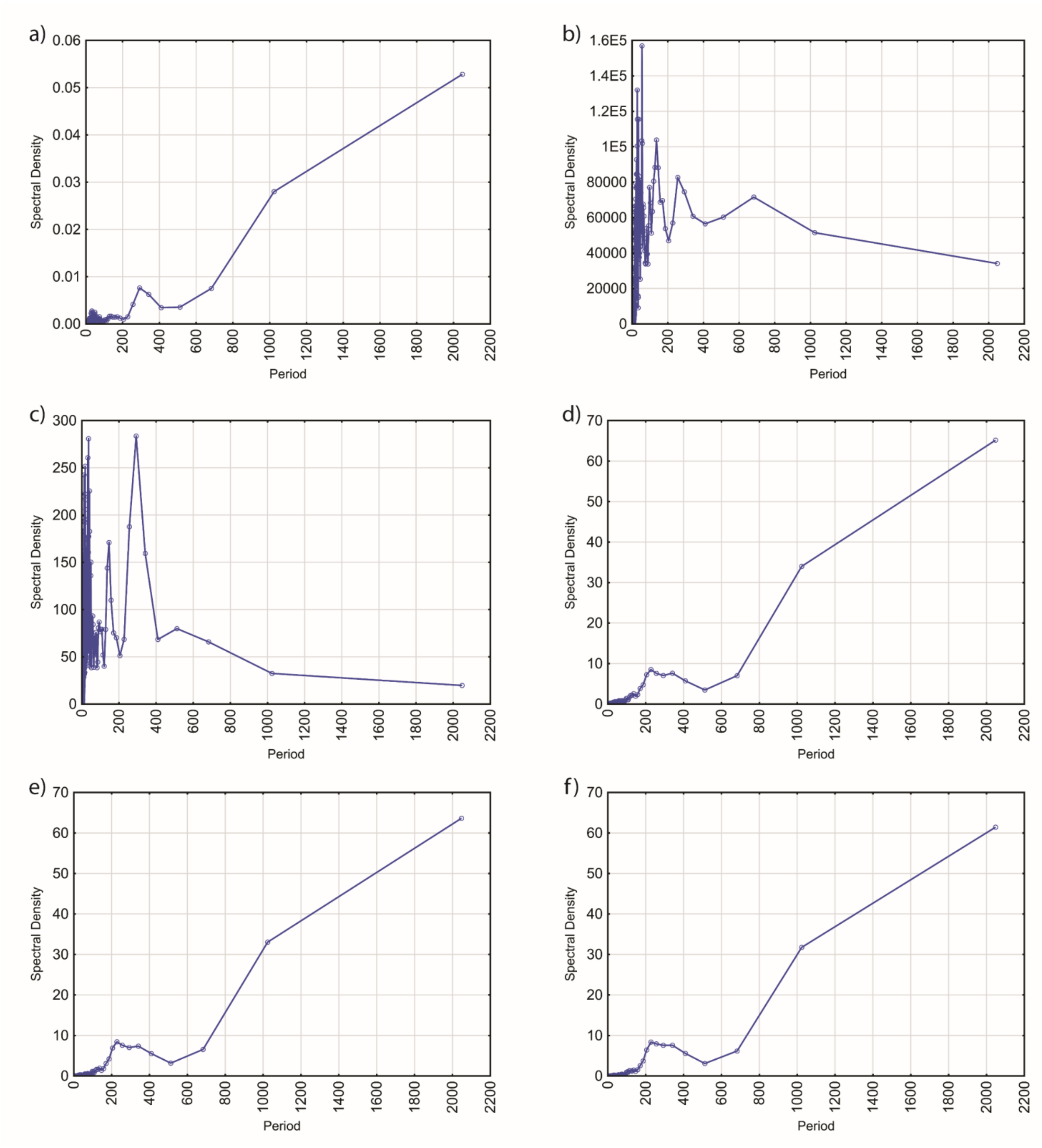

Fig. S1. Fourier spectral analysis results for winter-season measurements at Baldahin of (a) T, (b) CO_2, (c) visitors, (d) PM_{10}, (e) $PM_{2.5}$, (f) PM_1. Note that 288 units á 5 minutes equals 1 day cycle.

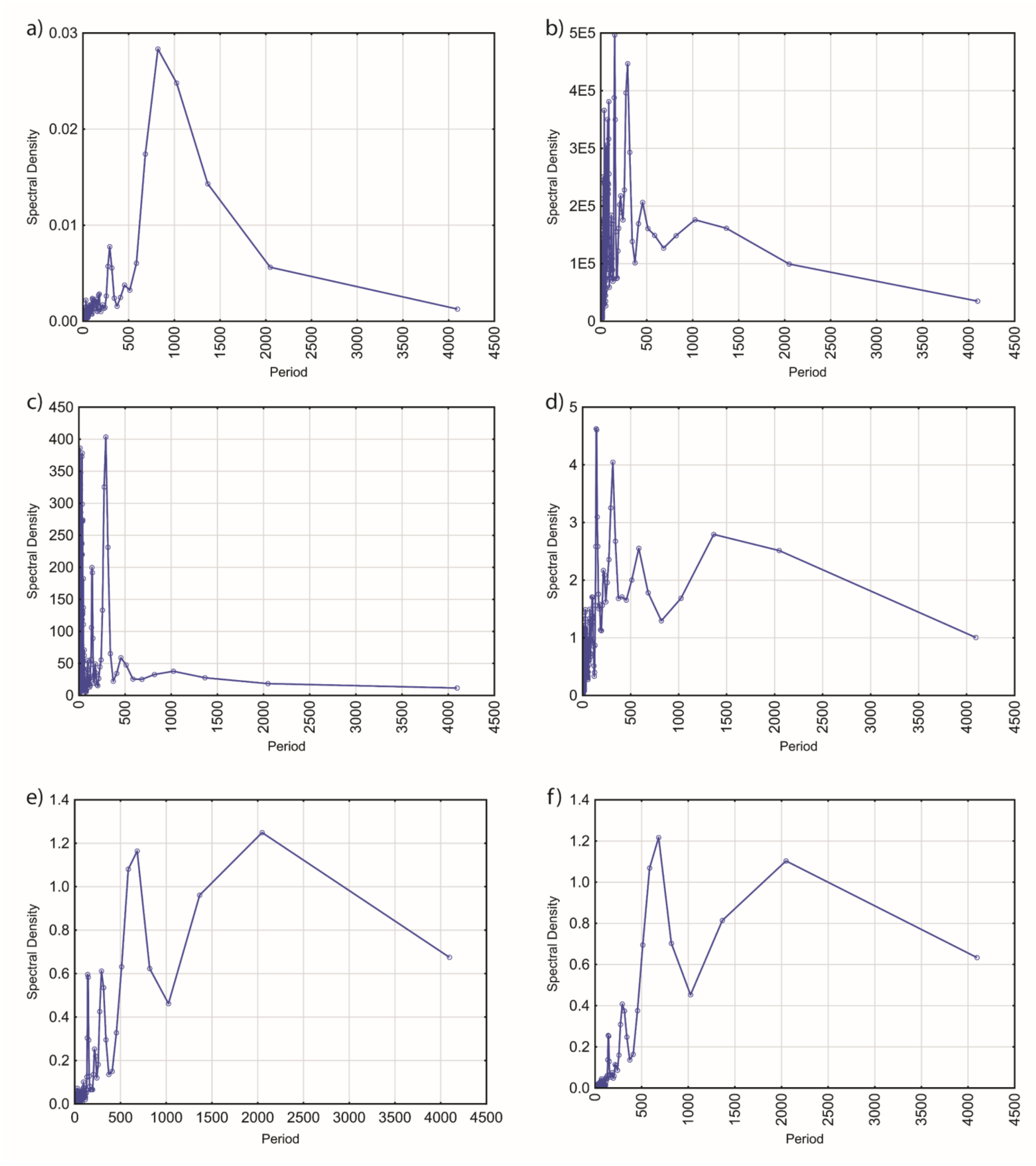

Fig. S2. Fourier spectral analysis results for winter-season measurements at Goba of (a) T, (b) CO_2, (c) visitors, (d) PM_{10}, (e) $PM_{2.5}$, (f) PM_1. Note that 288 units á 5 minutes equals 1 day cycle.

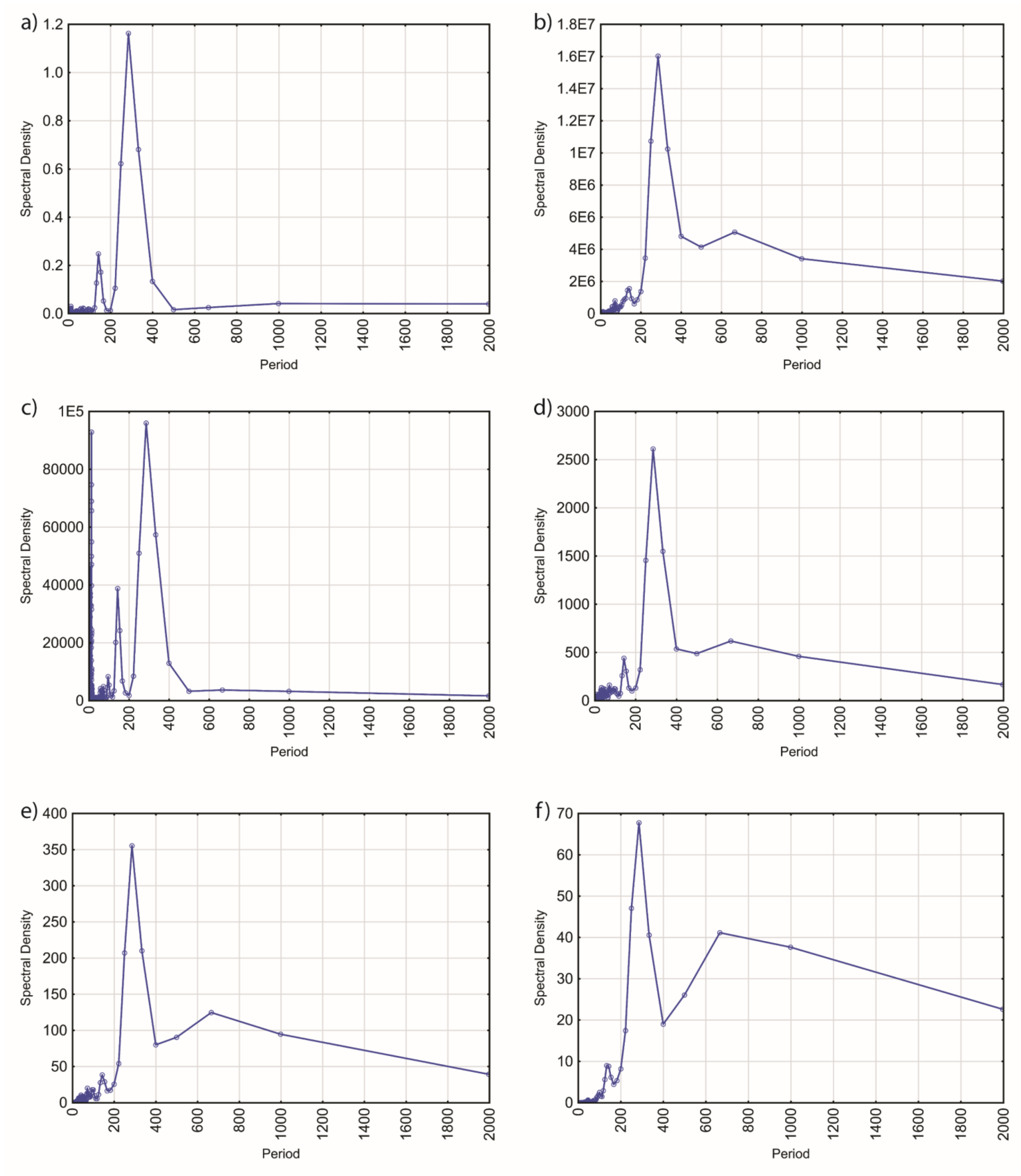

Fig. S3. Fourier spectral analysis results for summer-season measurements at Baldahin of (a) T, (b) CO_2, (c) visitors, (d) PM_{10}, (e) $PM_{2.5}$, (f) PM_1. Note that 288 units á 5 minutes equals 1 day cycle.

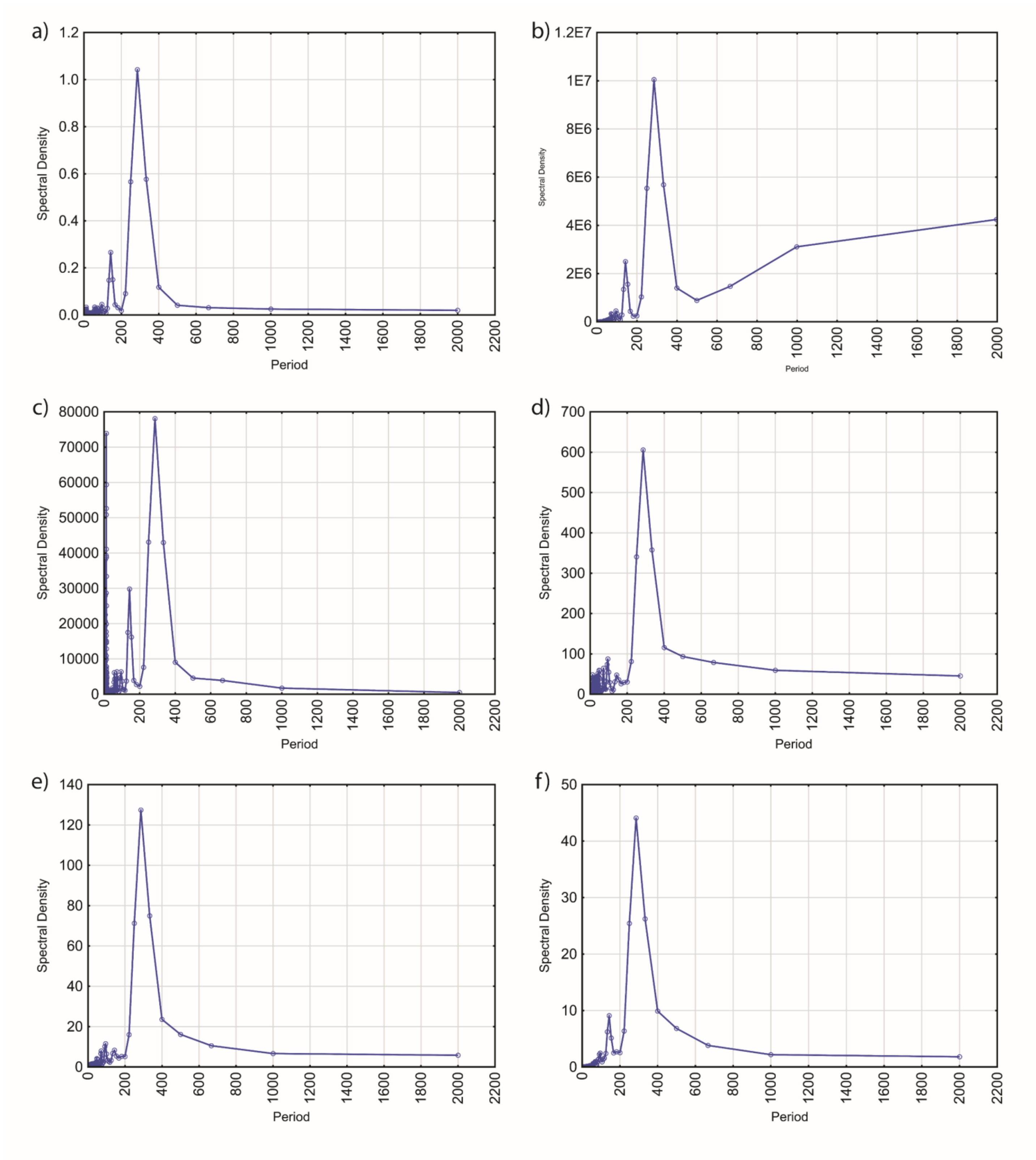

Fig. S4. Fourier spectral analysis results for summer-season measurements at Goba of (a) T, (b) CO_2, (c) visitors, (d) PM_{10}, (e) $PM_{2.5}$, (f) PM_1. Note that 288 units á 5 minutes equals 1 day cycle.

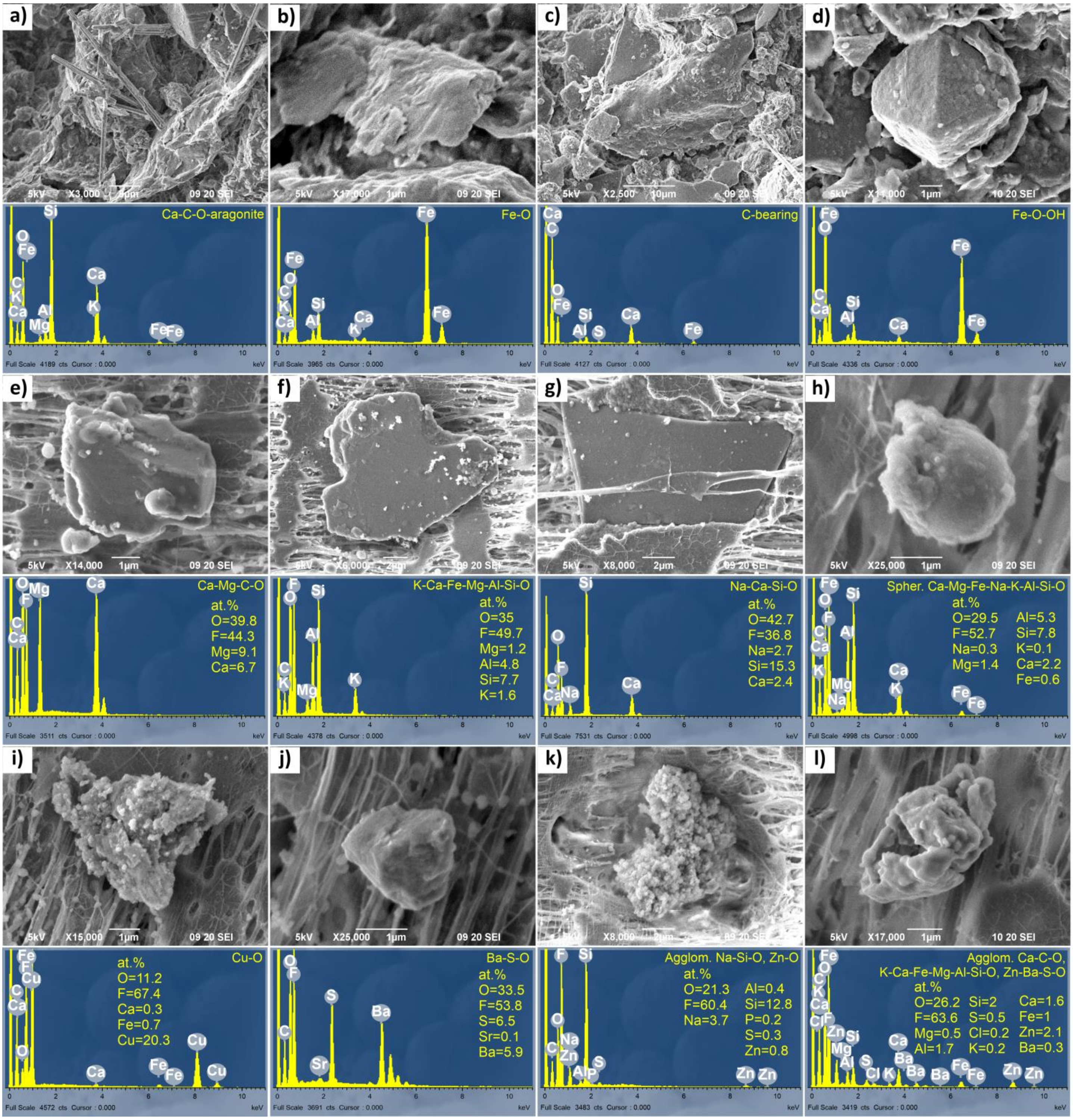

Fig. S5. SEM (BSE) images, EDS spectra, and semi-quantitative elemental composition of phases in cave sediment and PM: (a) Aragonite (Baldahin); (b) Fe-oxide shaving (Baldahin); (c) C-bearing particle (Baldahin); (d) Ferrihydrite or goethite (Goba); (e) Dolomite (Baldahin, winter); (f) Illite (Baldahin, winter); (g) Na-Ca silicate glass (Baldahin, winter); (h) Spherical Ca-Mg-Fe-Na-K-Al-Si-O (Baldahin, summer); (i) Cu-oxide (Goba, winter); (j) Barite (Goba, winter); (k) Agglomerate of Na-Si-O and Zn-oxide (Goba, summer); (l) Agglomerate of calcite, illite and Zn-Ba-S-O (Baldahin, summer). The samples are coated with carbon.
